# Detection of β-alanyl aminopeptidase as a biomarker for *Pseudomonas aeruginosa* in the sputum of patients with cystic fibrosis using exogenous volatile organic compound evolution

**DOI:** 10.1039/c9ra08386c

**Published:** 2020-03-12

**Authors:** Ryan Thompson, Dominic Stephenson, Hannah E. Sykes, John D. Perry, Stephen P. Stanforth, John R. Dean

**Affiliations:** Department of Applied Sciences, Northumbria University Ellison Building Newcastle upon Tyne NE1 8ST UK John.Dean@northumbria.ac.uk; Department of Microbiology, Freeman Hospital Newcastle upon Tyne NE7 7DN UK

## Abstract

A novel, rapid and sensitive analytical method has been developed and applied to 105 sputum samples from patients with cystic fibrosis, including 5 samples from post-lung transplant patients. This new method is specifically targeted to measure β-alanyl aminopeptidase activity which is characteristic of some important Gram-negative pathogens. Of relevance to this study are *Pseudomonas aeruginosa* and pathogens of the *Burkholderia cepacia* complex both of which are commonly associated with respiratory infections as well as increased morbidity and mortality in adult cystic fibrosis patients. The analytical method involves the addition of a novel enzyme substrate (*i.e.* 3-amino-*N*-(3-fluorophenyl)propanamide) that interacts with β-alanyl aminopeptidase to generate an exogenous volatile organic compound 3-fluoroaniline (LOD 0.02 μg mL^−1^; LOQ 0.06 μg mL^−1^). 3-Fluoroaniline was determined at 20 times above its calculated limit of quantification in the sputum samples by HS-SPME-GC-MS and then the results compared with standard culture methods and bacterial identification using MALDI-TOF-MS. Detection of 3-fluoroaniline was possible after only 8 h incubation of the sputum samples with a 95% success rate; this increased to 100% at 24 h which was well within the typical routine timeframe of 48 h. To our knowledge, this is the first demonstration of detection of *P. aeruginosa* by use of a custom-designed substrate to liberate a detectable and unique VOC. The very high negative predictive value (100% in this study) means such an assay could be appropriate as a screening technique for patients who are not yet colonized by this pathogen.

## Abstract 2

A novel, rapid and sensitive analytical method has been developed and applied to 105 sputum samples from patients with cystic fibrosis, including 5 samples from post-lung transplant patients.

## Graphical abstract

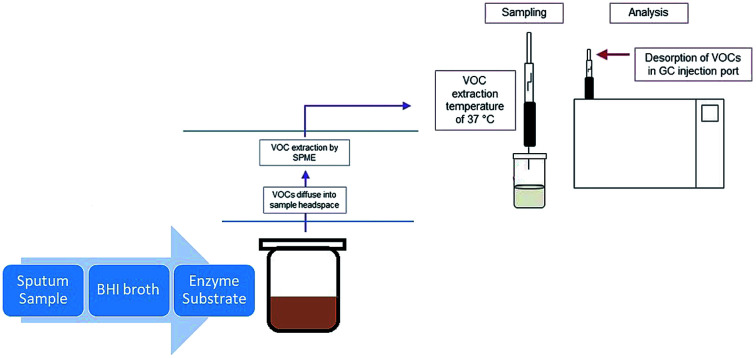

## Introduction

Cystic fibrosis (CF) was first identified as a distinct condition in 1938 by Dr Dorothy Anderson, who observed a pattern of cystic fibrosis of the pancreas in patients with a history of respiratory issues, subsequently leading to the identification of cystic fibrosis as a distinct condition.^1^ CF is a common autosomal recessive congenital disorder caused by mutations in the cystic fibrosis transmembrane conductance regulator (CFTR) gene responsible for anion transport, predominantly chloride & bicarbonate, across epithelial cell membranes and mucociliary clearance in respiratory airways.^2^

The absence or reduced function of the CFTR gene results in extra thick mucosal secretions in organ systems with epithelial cell linings, such as the lungs, digestive system, hepatobiliary tracts, and vas deferens in males.^3^ Due to this abnormally viscous lining, CF patients struggle to clear mucus from their airways and are very susceptible to further lung colonization and bacterial infections. In older children the first symptoms are often a mix of abnormal liver function test results, pancreatic exocrine insufficiency, an absence of vas deferens development in males, and persistent *Staphylococcus aureus* respiratory infections. As patients age into young adulthood, their symptoms often progress into cirrhosis of the liver due to chronic inflammation, intermittent respiratory infections from *Pseudomonas aeruginosa*, and established bronchiectasis. From approximately age 20 onwards, patients will frequently have established bronchiectasis associated with persistent respiratory infections with Gram-negative bacteria such as *Pseudomonas aeruginosa* and strains of the *Burkholderia cepacia* complex. The necessity for lung transplantation is frequently required for patients aged 30 onwards.^4^

Prognosis for CF patients is relatively poor in comparison to healthy individuals, although is improving along with better medical intervention. Based on data from the UK Cystic Fibrosis Registry, half of CF patients born in 2017 would have a life expectancy of approximately 47 years based upon current treatment and management regimes. However, in 2017 the median age of death for CF patients in the UK was reported as 31 years.^5^ The most common cause of death in CF patients is respiratory failure secondary to progressive lung disease, often caused by chronic infection. Due to an innate inability to clear the thick mucus associated with the condition, CF patients are susceptible to colonization by opportunistic pathogens that pose little risk to non-compromised individuals. The pathology associated with failure to clear microorganisms is exacerbated by the patient's immune response, causing chronic inflammation and tissue damage.^6^ In younger patients, the primary bacteria responsible for infection are *Staphylococcus aureus* and *Haemophilus influenzae*,^7^ both of which cause significant damage to the airways either directly, or indirectly through the immune inflammatory response.^8^ As the condition progresses, patients develop chronic bronchiectasis and become more susceptible to infection from Gram-negative bacteria commonly found in the environment, such as *Achromobacter* sp., *Stenotrophomonas maltophilia*, *Pseudomonas aeruginosa*, and *Burkholderia cepacia* complex strains.^9,10^ Additionally, there is an increase in prevalence of nontuberculous *Mycobacterium* infections in patients with cystic fibrosis, particularly *Mycobacterium abscessus* and *Mycobacterium avium-intracellulare*.^11,12^

*Pseudomonas aeruginosa* is by far the most common pathogen associated with respiratory infections in CF patients. In 2015, the UK national cystic fibrosis registry reported 49% of CF patients aged 18 and above had a chronic *P. aeruginosa* infection,^13^ however some studies report the incidence of *Pseudomonas* infection as high as 60% in adult patients.^14^ Some studies have shown that infection with *P. aeruginosa* can occur very early in childhood. In longitudinal studies on children diagnosed with CF, annual bronchoalveolar lavage cultures have shown a high prevalence of *P. aeruginosa* (∼11%).^7,15^ However, a further study which monitored the presence of *P. aeruginosa* antibodies found a higher incidence of infection at a younger age, as well as in patients who tested culture negative suggesting that some *P. aeruginosa* infections may be cleared naturally in younger patients.^16^ The early detection and treatment of *P. aeruginosa* infection is crucial and highly associated with a good prognosis. Early intervention with a strict antibiotic regime has been shown to achieve eradication of *P. aeruginosa* infection in approximately 60% of cases,^17^ however if this short window is missed, then infections are considered virtually impossible to eliminate.^17^ Chronic *P. aeruginosa* infections have clear associations with an accelerated decline in lung function,^18^ and increased mortality.^19^

Another important pathogen in CF patients are members of the *Burkholderia cepacia* complex, a group of 22 members of the *Burkholderia* genus. Infections from *B. cepacia* strains are strongly associated with a severe decline in lung function in CF patients, and an increased mortality rate.^20^ Patients infected with *B. cepacia* complex strains may also develop a life threatening systemic infection known as cepacia syndrome, where eradication of the infection is impossible and has been highly associated with post lung transplant mortality.^21^ The most common members of the *B. cepacia* complex strains associated with CF patients across Europe and Australasia are *B. multivorans* and *B. cenocepacia*, whereas in the United States and Canada, *B. multivorans* is the most prevalent.^20^ Interestingly, *Burkholderia gladioli*, a non *B. cepacia* complex strain member of the *Burkholderia* genus, has become more prominent in US CF patients, but is very uncommon elsewhere.^20^

*Pseudomonas* and *Burkholderia* infections are predominantly diagnosed in CF patients through the culturing of patient respiratory samples, such as sputum, cough swab, and bronchoalveolar lavages. Detection *via* culture relies on a plethora of different selective media, as there is no one definitive protocol for detection in hospitals so selective media panels and protocols are often developed in-house. Whilst culture methods have proved successful thus far for the isolation of *Pseudomonas* and *Burkholderia*, results are often only available following 48–72 h incubation, which when combined with the importance of beginning patient treatment early, could be detrimental to patient prognosis. The chromogenic culture medium CHROMID® *Pseudomonas* contains a chromogenic substrate for β-alanyl aminopeptidase, which is hydrolysed by *P. aeruginosa* resulting in the formation of purple colonies after 24 to 48 h of incubation.^22^ For specimens from cystic fibrosis patients, incubation can be extended up to 5 days. Our aim was to therefore develop a rapid test for the detection of *Pseudomonas* and *Burkholderia* from CF patient sputum samples, utilising β-alanyl aminopeptidase activity. We have previously identified that using targeted enzyme substrates, a unique exogenous volatile organic compound can be liberated and detected using either static headspace-solid phase micro-extraction-gas chromatography-mass spectrometry, SHS-SPME-GC-MS^23–26^ or static headspace multicapillary column-gas chromatography-ion mobility spectrometry, SHS-MCC-GC-IMS.^27^ The use of exogenous volatile organic compounds (VOCs) to distinguish 7 Gram-positive from 15 Gram-negative bacteria using the enzyme substrates 2-amino-*N*-phenylpropanamide (to liberate the VOC aniline) and 2-amino-*N*-(4-methylphenyl)propanamide (to liberate the VOC *p*-toluidine) has been demonstrated.^24^ Similarly the approach has been applied for the analysis of enzyme activity in *Listeria* species using the enzyme substrates benzyl-α-d-mannopyranoside (to liberate the VOC benzyl alcohol) and d-alanyl 3-fluoroanilide (to liberate the VOC 3-fluoroaniline).^25^ In addition, exogenous VOC detection has been used to identify *Salmonella* in milk samples using the enzyme substrates 2-chlorophenyl octanoate, phenyl α-d-galactopyranoside and L-pyrrolidonyl fluoroanilide which liberate the VOCs 2-chlorophenol, phenol and 3-fluoroaniline, respectively.^26^ In this case however, β-alanyl aminopeptidase enzymes work by catalysing the cleavage, *via* hydrolysis, of amino acids from the amino (N) terminus of protein or peptide substrates.^28^ The presence of β-alanyl aminopeptidase activity is well documented in *P. aeruginosa*,^29,30^ and has subsequently led to studies on the development of chromogenic and fluorogenic substrates targeting β-alanyl aminopeptidase activity as a method for the detection of *P. aeruginosa*.^31,32^ There is also further evidence for β-alanyl aminopeptidase activity in *Burkholderia cepacia* complex and *Serratia marcescens* in studies using the β-alanyl based 7-*N*-(β-alanyl)aminophenoxazin-3-one,^31^ 9-(4′-*N*-[β-alanyl]aminophenyl)acridines,^33^ and 9-(4′-*N*-[β-alanyl]aminophenyl)-10-methylacridinium salts.^34^

## Experimental

### Instrumentation and analysis

Gas chromatography-mass spectrometry (GC-MS) analyses were performed using a ThermoFinnigan Trace GC Ultra paired with a Polaris Q ion trap mass spectrometer (Thermo Fisher scientific, Loughborough, UK) with Xcalibur 1.4 SR1 software package (Thermo Fisher). Separation of the analytes was achieved using an Agilent Technologies (Wokingham, UK) DB-5MS column (30 m × 0.25 mm internal diameter × 0.25 μm film thickness), with the following temperature program: initial oven temperature 50 °C (hold 2 min), then a ramp to 250 °C @ 12.5 °C min^−1^, followed by a final hold time of 2 min. The Polaris Q ion trap mass spectrometer was set to full scan mode, scanning a mass range of 33–200 *m*/*z*, with a scan event time of 0.31 s. The ion source temperature was maintained at 260 °C, and the mass transfer line was maintained at 250 °C. Identification of VOCs was achieved using the National Institute of Standards and Technology (NIST) reference library (NIST Mass spectral library, version 2.0a, 2001) as well as the comparison of the retention times and mass spectra of authentic standards. All samples and standards were maintained at 37 °C during SPME sampling through the use of a water bath. All sputum samples were stored within a 37 °C incubator prior to analysis, and were placed in the water bath 10 min prior to SPME analysis using an 85 μm polyacrylate fibre (Sigma-Aldrich, Poole, UK) for temperature maintenance. Standards which were not previously stored at 37 °C were placed in the water bath for a minimum of 30 min prior to sampling. Fibres were used for analyte extraction with a manual holder, and were exposed in the headspace above all samples and standards for a total of 10 min. Following the adsorption of analytes, the fibre was immediately retracted inside the needle and transported directly to the inlet of the GC-MS. Analyte desorption was carried out by exposing the laden fibre within the split-splitless GC injection port at 250 °C for 2 min. The inlet was set to split mode with a split ratio of 1 : 10, with the helium carrier gas flow rate set to 1 mL min^−1^. Fibres were used for a maximum of 125 injections to ensure consistency of readings. The same SPME fibre type, method, and GC-MS program was used for all samples, blanks, and standards, throughout this study. The limit of detection (LOD) and limit of quantification (LOQ) for HS-SPME-GC-MS were determined by calculating the standard deviation (*n* = 7) of the background noise from the same retention time as the analyte. The LOD was determined by multiplying the standard deviation by 3, and the LOQ determined by multiplying by 10. Calibration curves were determined by running known concentrations of each exogenous VOC standard ranging from 0–100 μg mL^−1^, giving a *y* = *mx* + *c* value for sample concentration calculations. Due to variability of adsorptive efficiency within the fibres, a new calibration for each analyte was required each time a new fibre was used.

Matrix Assisted Laser Desorption Ionization-Time of Flight-Mass Spectrometry (MALDI-TOF-MS) analysis was performed by a Microflex LT mass spectrometer (Bruker Daltonik) using the MALDI Biotyper software package (version 3.0) with the reference database version 3.1.2.0. Isolated colonies were identified down to either genus or species level through the comparison of the sample generated spectra against that of an in-house taxonomy spectra library. All distinct colony morphologies isolated from the agar plates were subject to analysis *via* MALDI-TOF-MS by initially transferring a thin film of the colony onto a single stainless steel target plate position. The transferred colonies were covered with 1 μL of 70% formic acid then once dried, 1 μL HCCA matrix solution: alpha-cyano-4-hydroxycinnamic acid (10 mg HCCA dissolved in a solution of 475 μL distilled water, 500 μL acetonitrile, and 25 μL trifluoroacetic acid). Following the addition of the matrix solution, the samples were air dried prior to MALDI-TOF-MS analysis.

### Materials & reagents

Brain heart infusion broth (CM1136), brain heart infusion agar (CM1135), Columbia blood agar base (CM0331), CLED medium with Andrades indicator (CM0423), Sabouraud dextrose agar (CM0041), bacteriological agar no. 1 (LP001B), and Sputasol (SR0233A) were all purchased from Oxoid, Basingstoke, UK. Bacitracin (11072), chloramphenicol (C0378), Middlebrook 7H9 broth base (M0178), glycerol (G5516), 85 μm polyacrylate solidphase micro-extraction fibres, and *N*-methyl pyrrolidine (NMP) (328634) were purchased from Sigma, Gillingham, UK. Defribrinated horse blood (5% v/v, HB035) was purchased from TCS biosciences, Buckingham, UK. *Burkholderia cepacia* selective agar (PP0160) was purchased from EO laboratories, Bonnybridge, UK. Yeast extract (03904110) was purchased from bioMérieux, Marcy-l'Étoile, France. All bacteria used in this study were acquired from the Freeman Hospital Microbiology Department, Newcastle upon Tyne, UK. All *P. aeruginosa* strains (except NCTC 12903) were all strains isolated from patients with cystic fibrosis globally. All *Burkholderia* strains were part of the *Burkholderia cepacia* complex.

### Procedures for routine microbiological testing of cystic fibrosis patient samples

Patient sputum specimens are routinely received for culture by the Freeman Hospital Microbiology Department from clinics which are run for CF patients. The samples used in this study were aliquots of leftover samples referred to the Microbiology Department for routine analyses. No additional samples were taken for the purposes of this study. Samples were anonymized, except for the gender and age of the patient, by laboratory staff with legitimate access to patient data. Anonymization meant that patients could not be identified, and the results of this independent study could not be used to affect patient management in any way. Strict adherence to these conditions meant that patient consent was not sought, in accordance with the Medical Research Council Operational and Ethical Guidelines on the use of Human Tissue and Biological Samples for Use in Research.

Upon sample reception, the sputum specimens were mixed with an equal volume of sterile Sputasol and vortexed thoroughly until homogenization occurred. Homogenized samples were cultured onto 8 different agar media to ensure good differentiation and identification of organisms potentially present. Then, 1 mL aliquots of the homogenous specimens were transferred into sterile 20 mL glass vials and refrigerated at 4 °C until sample collection days.

#### Blood agar

Columbia blood agar base (Oxoid; CM0331, Basingstoke, UK) was mixed with distilled water to a concentration of 39 g L^−1^ and autoclaved at 121 °C for 15 min, following sterilisation 5% (v/v) defibrinated horse blood (TCS biosciences; HB035, Buckingham, UK) was added before dispensing approximately 20 mL per plate. Plates were streaked using a 10 μL loop. Plates were then incubated within a constant 5% CO_2_ atmosphere, and maintained at 37 °C.

#### Chocolate agar with bacitracin (CBAC)

Columbia blood agar base (Oxoid; CM0331, Basingstoke, UK) was mixed with distilled water to a concentration of 39 g L^−1^ and autoclaved. Following sterilisation, 5% (v/v) defibrinated horse blood (TCS biosciences; HB035, Buckingham, UK) was added, and the mixture was reheated to 80 °C to achieve haemolysis. Filter sterilised bacitracin (Sigma; 11702) dissolved in *N*-methyl 2-pyrrolidone (NMP) was then added to a final concentration of 70 mg L^−1^, and the mixture dispensed to approximately 20 mL per plate. Plates were streaked using a 10 μL loop. Plates were incubated within a constant 5% CO_2_ atmosphere, and maintained at 37 °C. Results were visually interpreted at both 24 and 48 h incubation.

#### Chocolate agar

Columbia blood agar base (Oxoid; CM0331, Basingstoke, UK) was mixed with distilled water to a concentration of 39 g L^−1^ and autoclaved. Following sterilisation, 5% (v/v) defibrinated horse blood (TCS biosciences; HB035, Buckingham, UK) was added, and the mixture was reheated to 80 °C to achieve haemolysis. The mixture was then dispensed to approximately 20 mL per plate. Plates were inoculated by first inoculating 2 mL of sterile water with 1 μL loop of sputum sample, from this inoculated water a 10 μL loop was used for streaking. Plates were incubated within a constant 5% CO_2_ atmosphere, and maintained at 37 °C. Results were visually interpreted at both 24 and 48 h incubation.

#### Cystine–lactose–electrolyte deficient agar (CLED)

CLED medium with Andrades indicator (Oxoid; CM0423, Basingstoke, UK) was mixed with distilled water to 36.2 g L^−1^ and autoclaved. Following sterilisation, the mixture was dispensed as standard. Plates were streaked using a 10 μL loop. Plates were incubated within a non-modified air atmosphere and maintained at 37 °C. Results were visually interpreted at both 24 and 48 h incubation.

#### Sabouraud agar with chloramphenicol (SAB)

Sabouraud dextrose agar (Oxoid; CM0041, Basingstoke, UK) was mixed with distilled water to 65 g L^−1^ and autoclaved. Following autoclaving, filter sterilised chloramphenicol heated at 100 °C for 5 min (Sigma; C0378) was added to the mixture at 50 mg L^−1^, and the mixture dispensed. Plates were streaked using a 10 μL loop. Plates were incubated within a non-modified air atmosphere and maintained at 37 °C. Results were visually interpreted following 5 days of incubation.

#### Aztreonam selective agar (AZTRE)

Columbia blood agar base (Oxoid; CM0331, Basingstoke, UK) was mixed with distilled water to a concentration of 39 g L^−1^ and autoclaved at 121 °C for 15 min, following sterilisation 5% (v/v) defibrinated horse blood (TCS biosciences; HB035, Buckingham, UK) was added. Filter sterilised aztreonam (Azactam) was added to the mixture at 15.625 mg L^−1^ before dispensing. Plates were streaked using a 10 μL loop. Plates were incubated within a non-modified air atmosphere, and maintained at 37 °C. Results were visually interpreted at both 24 and 48 h incubation.

#### *Burkholderia cepacia* agar (CEP)

*Burkholderia cepacia* selective agar was purchased ready-made (E&O labs, PP0160, Bonnybridge, UK). Plates were inoculated through the filling of a 3 mL pastette with the sputum sample, and subsequently streaking 2 drops. Plates were incubated within a non-modified air atmosphere, and maintained at 37 °C. Results were visually interpreted following 5 days of incubation.

#### Rapidly growing mycobacterium agar (RGM)

RGM agar was prepared as previously described.^35^

### Individual substrate testing against pure *Pseudomonas aeruginosa* and *Burkholderia cepacia* complex strains

Sample vials were prepared by first suspending bacterial cells in sterile Brain Heart Infusion (BHI) broth and adjusting the absorbance to 0.132 when measured at 600 nm. This gave an inoculum of approximately 1–1.5 × 10^8^ CFU mL^−1^, equivalent to 0.5 McFarland units. The sterile BHI broth was prepared by dissolving 37 g of the powder mix into 1 litre of MilliQ water, and sterilising the resulting mix *via* autoclave at 121 °C for 15 min. Following this, 100 μL of 1 × 10^8^ CFU mL^−1^ stock was added to 9.89 mL of sterile BHI broth. To this new bacterial suspension, 10 μL of the chosen substrate *i.e.* 3-amino-*N*-(3-fluorophenyl)propanamide, 3-amino-*N*-(4-methylphenyl)propanamide or 3-amino-*N*-phenylpropanamide (dissolved in NMP to 100 mg mL^−1^) was added. The final volume of the sample vials was 10 mL, with a final bacterial inoculum of 1 × 10^6^ CFU mL^−1^, and a final substrate concentration of 100 μg mL^−1^. Sterile 20 mL glass vials with septum lids were used for all samples. Once prepared, the sample vials were placed into an incubator set to 37 °C overnight, ranging from 20–24 h. Following overnight incubation, the samples were then analysed *via* HS-SPME-GC-MS.

### Procedure for analysis of cystic fibrosis patient sputum samples by HS-SPME-GC-MS

On sample collection days, 10 mL of BHI broth containing 100 μg mL^−1^ of 3-amino-*N*-(3-fluorophenyl)propenamide, 5 mg L^−1^ amphotericin B (an anti-fungal) and 70 mg L^−1^ bacitracin (a combination of peptides that inhibit the growth of yeasts and Gram-positive bacteria) was added to sample vials containing 1 mL specimen and then vortexed to thoroughly mix. Samples were then collected and transported to the University Laboratories (within 1 h) where they were incubated at 37 °C for set time periods before analysis. Great care was taken to not disturb or move the lids of the vials. Samples were placed into the incubator in 20 min intervals, to account for the GC-MS runtime and ensure that all samples were analysed following either 8 h or 24 h incubation.

### Procedures for synthesis of enzyme substrates

The β-alanyl substrates 1a–c [as their trifluoroacetate (TFA) salts] were prepared from their corresponding Boc-protected derivatives 2a–c.
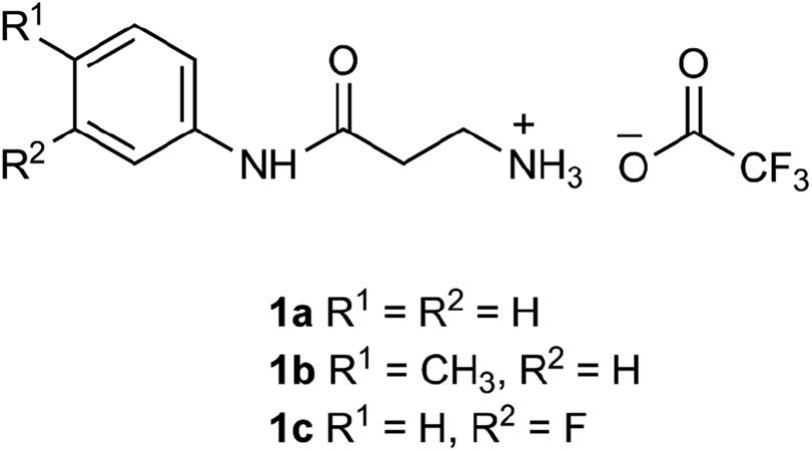


Compounds 2a–c were synthesised from β-alanyl and an appropriate aniline derivative using a mixed anhydride coupling reaction. *tert*-Butyl (3-anilino-3-oxopropyl)carbamate 2a and 3-amino-*N*-phenylpropanamide (TFA salt) 1a have been described previously.^36^
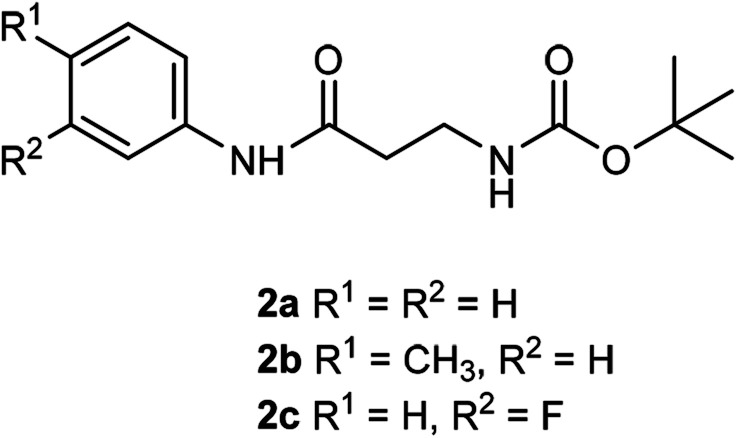


### General procedure for mixed anhydride coupling

To Boc-β-alanyl (1.2 equivalents) in dry THF, *N*-methylmorpholine (1.05 equivalents) was added and reaction mixture was cooled to −10 °C and then stirred for 30 min. Isobutyl chloroformate (1.05 equivalent) was then added, the mixture was stirred (2 min) and then a pre-cooled (−10 °C) solution of the appropriate aniline (1 equivalent) dissolved in THF (8 mL) was added drop-wise. The mixture was stirred (2 h) at −10 °C, allowed to warm to room temperature and then stirred overnight. The mixture was concentrated and water was added to the residue. The mixture was extracted with CH_2_Cl_2_ (2 × 20 mL) and the combined organic extracts were washed sequentially with aqueous citric acid solution (0.1 M, 2 × 20 mL), aqueous NaHCO_3 solution_ (10%, 2 × 20 mL), water (2 × 20 mL), dried (MgSO_4_) and concentrated to give the crude product. Recrystallisation from methanol gave pure compounds as white crystals.

### General procedure for Boc-deprotection

The appropriate Boc-protected substrate 2a–c was suspended in dry CH_2_Cl_2_ and then trifluoroacetic acid was added dropwise. The resulting mixture was stirred (4 h) at room temperature and evaporated. The resulting oil was triturated with diethyl ether until a precipitate formed. The solid was collected and washed with diethyl ether giving the desired products (as their TFA salts) as white, hydroscopic solids.

### Analytical data

#### 3-Amino-*N*-(4-methylphenyl)propanamide, TFA salt 1b

^1^H-NMR (400 MHz; DMSO-d_6_) *δ*: 10.1 (1H, s, N–H), 7.93 (3H, broad s, –NH_3_^+^), 7.44 (2H, d, *J* = 8.8 Hz, Ar–H), 7.06 (2H, d, *J* = 8.8 Hz, Ar–H), 3.04 (2H, m, CH_2_), 2.66 (3H, t, *J* = 6.8 Hz, CH_2_), 2.2 (3H, s, CH_3_); ^13^C-NMR (101 MHz; DMSO-d_6_) *δ*: 168.6 (C=O), 159.1 (q, *J* = 31 Hz, TFA), 136.9 (Ar–C), 132.7 (Ar–C), 129.6 (2 × Ar–C), 119.9 (2 × Ar–C), 118.9 (q, *J* = 297 Hz, TFA), 35.5 (CH_2_), 33.7 (CH_2_), 20.9 (CH_3_); HRMS: *m*/*z* found, 179.1177. C_10_H_15_N_2_O [MH]^+^ requires, 179.1184.

#### 3-Amino-*N*-(3-fluorophenyl)propanamide, TFA salt 1c

^1^H-NMR (400 MHz; DMSO-d_6_) *δ*: 10.42 (1H, s, N–H), 7.88 (3H, broad s, –NH_3_^+^), 7.57 (1H, d, *J* = 11.6 Hz, Ar–H), 7.31–7.26 (2H, m, Ar–C), 6.84 (1H, t, *J* = 7.6 Hz, Ar–H), 3.05 (2H, broad s, CH_2_), 2.68 (2H, t, *J* = 6.8 Hz, CH_2_); ^13^C-NMR (101 MHz; DMSO-d_6_) *δ*: 169.3 (C=O), 162.6 (d, *J* = 240 Hz, Ar–C), 141.1 (d, *J* = 10 Hz, Ar–C), 130.9 (d, *J* = 10 Hz, Ar–C),115.4 (Ar–C), 110.3 (d, *J* = 21 Hz, Ar–C), 106.5 (d, *J* = 27 Hz, Ar–C), 35.3 (CH_2_), 33.8 (CH_2_); HRMS: *m*/*z* found, 183.0929. C_9_H_12_N_2_OF [MH]^+^ requires, 183.0934.

#### *tert*-Butyl [3-(4-methylanilino)-3-oxopropyl]carbamate 2b

^1^H-NMR (400 MHz; DMSO-d_6_) *δ*: 9.80 (1H, broad s, *N*–H), 7.42 (2H, d, *J* = 8.8 Hz, Ar–H), 7.03 (2H, d, *J* = 8.8 Hz, Ar–H), 6.82 (1H, broad s, N–H), 3.16 (2H, dt, *J* = 6.9 and 6.9 Hz, CH_2_), 2.39 (2H, t, *J* = 6.9 Hz, CH_2_), 2.19 (3H, s, CH_3_), 1.33 (9H, s, (CH_3_)_3_); ^13^C-NMR (101 MHz; DMSO-d_6_) *δ*: 169.6 (C=O), 156.1 (C=O), 137.2 (Ar–C), 132.4 (Ar–C), 129.5 (2 × Ar–C), 119.6 (2 × Ar–C), 78.1 (C-Bu^*t*^), 37.2 (CH_2_), 37.1 (CH_2_), 28.8 (3 × CH_3_), 21.0 (CH_3_).

#### *tert*-Butyl [3-(3-fluoroanilino)-3-oxopropyl]carbamate 2c

^1^H-NMR (400 MHz; DMSO-d_6_) *δ*: 10.11 (1H, broad s, N–H), 7.57 (1H, d, *J* = 12.0 Hz, Ar–H), 7.28–7.25 (2H, m, Ar–H), 6.85–6.80 (2H, m, Ar–H and N–H), 3.18 (2H, dt, *J* = 7.2 and 7.2 Hz, CH_2_), 2.44 (2H, t, *J* = 7.2 Hz, CH_2_), 1.33 (9H, s, (CH_3_)_3_); ^13^C-NMR (101 MHz; DMSO-d_6_) *δ*: 170.3 (C=O), 162.6 (d, *J* = 239 Hz, Ar–C), 156.1 (C=O), 141.4 (d, *J* = 11 Hz, Ar–C), 130.7 (d, *J* = 10 Hz, Ar–C), 115.3 (Ar–C), 109.9 (d, *J* = 21 Hz, Ar–C), 106.3 (d, *J* = 26 Hz, Ar–C), 78.2 (C-Bu^*t*^), 37.3 (CH_2_), 36.9 (CH_2_), 28.7 (3 × CH_3_).

## Results & discussion

Analytical data was determined for all three tested exogenous volatile organic compound standards *i.e.* aniline, 3-fluoroaniline and *p*-toluidine, by HS-SPME-GC-MS. Calibration graphs were also obtained for all three VOCs over the concentration range 0–100 μg mL^−1^ with correlation coefficients, *R*^2^, of >0.99 in all cases. The limit of detection (LOD) and limit of quantitation (LOQ), based on 3 or 10× standard deviations of the blank, were determined for each VOC (Table 1). The inclusion of a pre-concentration step, using SPME, allowed excellent LOD and LOQ to be obtained using GC-MS (Table 1), typically 0.02 μg mL^−1^ and 0.06 μg mL^−1^ for 3-fluoroaniline.

**Table 1:** Analytical data for the quantification of exogenous volatile organic compounds by HS-SPME-GC-MS for analysis of sputum samples from cystic fibrosis patients

VOC	GC retention time (mins)	Quantitative *m*/*z*	Linear range (μg mL^−1^)	*y* = *mx* + *c*	*R* ^2^	*n*	LOD (μg mL^−1^)	LOQ (μg mL^−1^)
Aniline	6.2	39–66–93	0–100	*y* = 5146.7 + 83.4	0.9970	5	0.15	0.37
3-Fluoroaniline^a^	6.7	83–84–111	0–100	*y* = 18 337*x* + 314.2	0.9970	5	0.02	0.06
*p*-Toluidine	7.6	77–106–107	0–100	*y* = 11 163*x* + 417.3	0.9970	5	0.09	0.24

a Three 85 μm polyacrylate SPME fibres were used during the analysis of cystic fibrosis patient sputum samples. Previous use of SPME technology identified that fibre degradation does not occur until around 200 injections; in this study SPME fibres were used for a maximum of 125 injections to ensure consistency between samples. Due to variability of adsorptive efficiency within the SPME, a new calibration for 3-fluoroaniline was generated each time a new fibre was used; in addition, the LOD and LOQ was also re-calculated for each fibre. The analytical data obtained was as follows: SPME fibre 1: *y* = 18 694*x* + 308.5, *R*^2^ = 0.9970, LOD 0.017 μg mL^−1^ and LOQ = 0.057 μg mL; SPME fibre 2: *y* = 18 374*x* + 254.9, *R*^2^ = 0.9960, LOD 0.017 μg mL^−1^ and LOQ = 0.057 μg mL; and, SPME fibre 3: *y* = 18 136*x* + 267, *R*^2^ = 0.9970, LOD 0.015 μg mL^−1^ and LOQ = 0.049 μg mL^−1^.

Initially, the three potential enzyme substrates were tested against a panel of 12 *P. aeruginosa* strains and 12 *Burkholderia cepacia* complex strains (Table 2). All bacteria were tested in BHI broth at a pre-incubation inoculum of 1 × 10^6^ CFU mL^−1^, and substrates were added at 100 μg mL^−1^, equivalent to 1.07 mmol L^−1^ aniline, 1.11 mmol L^−1^ 3-fluoroaniline and 0.93 mmol L^−1^*p*-toluidine, using NMP as the solvent. All samples were incubated overnight at 37 °C prior to sampling *via* HS-SPME-GC-MS. Good activity was observed (typically > 20 μg mL^−1^ VOC liberated) for all *P. aeruginosa* strains across all 3 β-alanyl aminopeptidase substrates. This supports the previous findings in the literature regarding activity in this species.^29,30^ There was some variation in enzyme activity between the *P. aeruginosa* strains, for instance using substrate 3-amino-*N*-(3-fluorophenyl)propanamide the AA44 strain produced an average of 38.5 μg mL^−1^ (or 346 μmol L^−1^) of its VOC product (3-fluoroaniline), whereas strain AUS52 produced an average of 19.8 μg mL^−1^ (or 178 μmol L^−1^). In general terms, the 3-amino-*N*-(4-methylphenyl)propanamide substrate evolved its VOC (*i.e. p*-toluidine) almost always to a lower concentration (Table 2).

**Table 2:** Preliminary β-alanyl substrate testing of *P. aeruginosa* strains and *B. cepacia* complex strains

ID number	Strain ID	β-Alanyl substrates					
		Substrate: 3-amino-*N*-(3-fluorophenyl)propanamide		Substrate: 3-amino-*N*-(4-methylphenyl) propanamide		Substrate: 3-amino-*N*-phenylpropanamide	
		VOC: 3-fluoroaniline		VOC: *p*-toluidine		VOC: aniline	
		Growth	μg mL^−1^	Growth	μg mL^−1^	Growth	μg mL^−1^
LMG 27632	*P. aeruginosa* AA44	+	**38.5** (37.6, 39.3)	+	**21.0** (20.9, 21.2)	+	**36.4** (37.1, 35.8)
LMG 27631	*P. aeruginosa* AA43	+	**33.4** (34.5, 32.4)	+	**22.0** (21.4, 22.6)	+	**27.3** (28.2, 26.5)
LMG 27629	*P. aeruginosa* AUS52	+	**19.8** (19.8, 19.7)	+	**20.8** (20.1, 21.5)	+	**20.0** (20.3, 19.8)
LMG 27638	*P. aeruginosa* PA01	+	**32.7** (32.3, 33.1)	+	**17.5** (17.5, 17.4)	+	**28.6** (28.8, 28.5)
LMG 27626	*P. aeruginosa* DK2	+	**35.6** (35.4, 35.7)	+	**21.3** (21.2, 21.5)	+	**28.4** (27.4, 29.3)
LMG 27624	*P. aeruginosa* LES431	+	**35.5** (36.3, 34.8)	+	**19.7** (19.3, 20.2)	+	**26.5** (26.0, 26.9)
LMG 27627	*P. aeruginosa* AES1R	+	**26.4** (25.6, 27.2)	+	**19.5** (19.1, 20.0)	+	**25.9** (26.4, 25.4)
LMG 27630	*P. aeruginosa* AA2	+	**26.0** (25.9, 26.1)	+	**17.9** (18.1, 17.7)	+	**22.9** (23.6, 22.2)
LMG 27622	*P. aeruginosa* LESB58	+	**24.9** (24.7, 25.1)	+	**21.1** (21.4, 20.7)	+	**28.4** (27.7, 29.1)
LMG 27625	*P. aeruginosa* C3719	+	**23.9** (23.4, 24.3)	+	**18.4** (18.9, 17.9)	+	**22.5** (23.0, 21.9)
LMG 27623	*P. aeruginosa* LES400	+	**20.7** (20.8, 20.6)	+	**18.0** (17.9, 18.2)	+	**22.1** (21.8, 22.4)
NCTC 12903	*P. aeruginosa* NCTC 12903	+	**21.2** (20.7, 21.2)	+	**18.3** (17.9, 18.7)	+	**22.6** (23.1, 22.1)
LMG 18824	*B. multivorans*	+	**1.2** (1.2, 1.2)	+	**2.5** (2.5, 2.5)	+	**1.0** (1.0, 1.0)
LMG 14294	*B. stabilis*	+	**8.4** (8.2, 8.6)	+	**15.7** (15.4, 15.9)	+	**6.8** (6.6, 7.0)
LMG 18828	*B. cenocepacia*	+	**2.3** (2.3, 2.2)	+	**4.4** (4.4, 4.4)	+	**2.6** (2.5, 2.6)
LMG 18822	*B. multivorans*	+	**1.8** (1.8, 1.9)	+	**3.8** (3.9, 3.8)	+	**2.3** (2.3, 2.3)
LMG 18821	*B. cepacia*	+	**2.4** (2.4, 2.4)	+	**4.1** (4.1, 4.2)	+	**2.2** (2.2, 2.1)
LMG 16656	*B. cenocepacia*	+	**7.9** (8.1, 7.7)	+	**10.3** (10.4, 10.2)	+	**5.8** (5.8, 5.8)
LMG 10929	*B. vietnamiensis*	+	0.0 (0.0, 0.0)	+	0.0 (0.0, 0.0)	+	0.0 (0.0, 0.0)
LMG 17588	*B. multivorans*	+	**12.8** (12.5, 13.2)	+	**21.0** (21.1, 20.9)	+	**10.6** (10.8, 10.5)
LMG 16660	*B. multivorans*	+	**1.5** (1.4, 1.5)	+	**3.4** (3.3, 3.4)	+	**2.3** (2.3, 2.3)
LMG 13010	*B. multivorans*	+	**2.8** (2.8, 2.8)	+	**8.9** (8.7, 9.2)	+	**5.5** (5.2, 5.9)
LMG 16232	*B. vietnamiensis*	+	0.0 (0.0, 0.0)	+	0.0 (0.0, 0.0)	+	0.0 (0.0, 0.0)
LMG 1222	*B. cepacia*	+	**1.7** (1.7, 1.7)	+	**5.0** (4.9, 5.1)	+	**3.1** (3.0, 3.1)

Enzyme activity was markedly less substantial in the *B. cepacia* complex strains than in *P. aeruginosa* but was still significant enough for straightforward quantification. *B. multivorans*, *B. cenocepacia*, *B. cepacia*, and *B. stabilis* all gave positive results for β-alanyl aminopeptidase activity, with only *B. vietnamiensis* testing negative in LMG strains 10 929 and 16 232 across all substrates. As was observed in the *P. aeruginosa* strains, there was significant variability in enzyme activity between members of the same species when using the same substrate. For instance, *B. multivorans* was the most tested member of the *B. cepacia* complex strains with 5 strains tested, and when tested with substrate 3-amino-*N*-(3-fluorophenyl)propanamide produced average VOC concentrations of between 1.2 μg mL^−1^ and 12.8 μg mL^−1^. Overall, the results from the β-alanyl substrates were deemed to be acceptable (Table 2) despite the lack of activity in *B. vietnamiensis*, however this is somewhat offset given the minimal role *B. vietnamiensis* plays in cystic fibrosis associated infections in comparison to *B. multivorans*, *B. cenocepacia*, and *B. cepacia*.^37^

In order to ascertain which substrate would be most suitable for further testing, a statistical analysis on the performance of each β-alanyl substrate amongst the *P. aeruginosa* strains was performed. A single factor ANOVA, with a *p*-value of 0.0002 (*p* ≤ 0.05) was performed which indicated there was a statistically significant difference between the mean values of enzyme activity, as assessed by the VOC concentration, between the 3 substrates. However, an ANOVA does not give an indication to where the difference between the groups lies, and therefore requires individual *T*-tests between paired groups to determine which substrate produced the best results. Prior to *T*-tests, *F*-tests were performed to determine equal or unequal variance between the paired sample groups. An initial *F*-test was conducted between the data from substrates 3-amino-*N*-(3-fluorophenyl)propanamide and 3-amino-*N*-(4-methylphenyl)propanamide, which gave an *F* value of 17.4, and an *F* critical value of 2.8; as the *F* value was greater than the *F* critical value, we can reject the null hypothesis that sample variance is equal between these datasets, and accept the alternative hypothesis that the variance is unequal. Next, an *F*-test was carried out for the data acquired from 3-amino-*N*-(3-fluorophenyl)propanamide and 3-amino-*N*-phenylpropanamide, giving subsequent *F* and *F* critical values of 2.2 and 2.8, respectively, therefore indicating equal variance between the two datasets. Finally, another *F*-test was conducted between substrates 3-amino-*N*-(4-methylphenyl)propanamide and 3-amino-*N*-phenylpropanamide, giving subsequent *F* and *F* critical values of 7.8 and 2.8, respectively, therefore indicating unequal variance between the two datasets.

Following the implementation of *F*-test analyses, *T*-test analyses were then performed on paired data sets. Firstly, a *T*-test assuming unequal sample variances was conducted between 3-amino-*N*-(3-fluorophenyl)propanamide and 3-amino-*N*-(4-methylphenyl)propanamide, giving a two tail *p*-value of 0.0009 meaning there is a statistically significant difference (*p* < 0.05) between the means of the two datasets, with 3-amino-*N*-(3-fluorophenyl)propanamide producing a higher concentration of VOC product from enzyme activity. Next a *T*-test assuming equal sample variance was performed between 3-amino-*N*-(3-fluorophenyl)propanamide and 3-amino-*N*-phenylpropanamide, gave a two tail *p*-value of 0.3350 (*p* < 0.05), meaning that no statistically significant difference can be inferred between these two datasets, and that the substrates behave in a very similar manner in terms of VOC concentration evolution. Lastly, a *T*-test assuming unequal samples variances was carried out between 3-amino-*N*-(4-methylphenyl)propanamide and 3-amino-*N*-phenylpropanamide, which gave a two tail *p*-value of 0.0003 (*p* < 0.05), meaning there was a statistically significant difference between the average VOC concentration evolution in our 12 *P. aeruginosa* strains between these two substrates, with 3-amino-*N*-phenylpropanamide the more productive of the two.

The results of all the *T*-test analyses indicated that whilst 3-amino-*N*-(4-methylphenyl)-propanamide performed the poorest out of the three β-alanyl aminopeptidase substrates, there was no statistically significant difference between the performance of 3-amino-*N*-(3-fluorophenyl)propanamide and 3-amino-*N*-phenylpropanamide. However, from the initial calibration data in Table 1, for the two VOCs produced by these compounds (3-fluoroaniline by 3-amino-*N*-(3-fluorophenyl)propanamide, and aniline by 3-amino-*N*-phenylpropanamide), the HS-SPME-GC-MS method gave a lower limit of detection and limit of quantification for 3-fluoroaniline than aniline; therefore making 3-amino-*N*-(3-fluorophenyl)propanamide the most suitable enzyme substrate for subsequent testing.

**Fig. 1:**
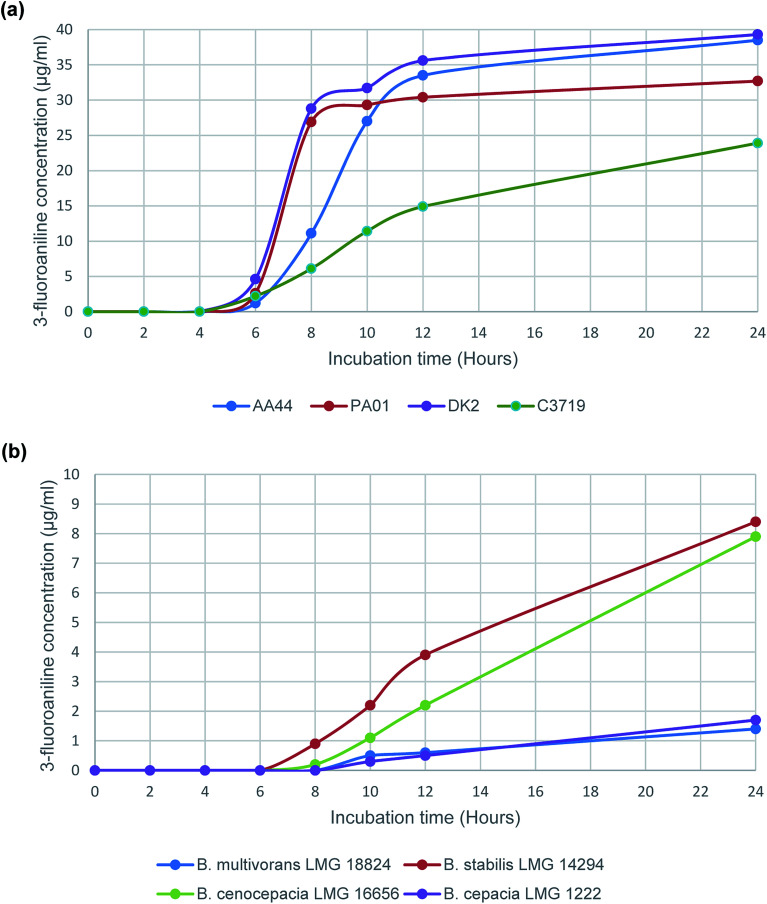
Evolution of 3-fluoroaniline using the enzyme substrate 3-amino-*N*-(3-fluorophenyl)propanamide with selected (a) *P. aeruginosa* strains and (b) *B. cepacia* complex strains.

Subsequently a time study was performed using the enzyme substrate, 3-amino-*N*-(3-fluorophenyl)propanamide, to determine 3-fluoroaniline evolution from a panel of 4 randomly selected *P. aeruginosa* strains and 4 strains that represent the sub-species of the *B. cepacia* complex. The results (Fig. 1), from the time study on *P. aeruginosa* strains, were consistent (Fig. 1(a)), with increasing VOC evolution observed after 6 h of incubation in all tested strains; typical 3-fluoroaniline concentrations ranging from 1.2 μg mL^−1^ to 4.6 μg mL^−1^, which was at least 20 times higher than the estimated LOQ (Table 1). Results for the *B. cepacia* complex strains were understandably less impressive than those of the *P. aeruginosa* strains. None of the 4 strains were able to produce any VOC evolution following 6 h of incubation, and only 2 of the 4 strains tested were able to produce a detectable VOC signal following 8 h of incubation (*B. stabilis* and *B. cenocepacia*). However, all 4 of the tested strains were able to produce the VOC following 10 h of incubation; typical 3-fluoroaniline concentrations ranging from 0.3 μg mL^−1^ to 2.2 μg mL^−1^, which was at least 5 times higher than the estimated LOQ (Table 1).

### Detection of *P. aeruginosa* strains and *B. cepacia* complex strains in sputum from patients with cystic fibrosis

A blind study was undertaken of 100 sputum samples collected and analysed over the period of one calendar month from 78 distinct CF patients (49 male : 51 female). In addition, a further 5 samples were analyzed from CF patients who were post-lung transplant. All generated data was evaluated post-analyses (Table 3). The data identified that no false negative samples were observed in the sputum sample data set. However, there were 7 instances in which initial consideration of the exogenous VOC data concluded a false positive result based on identification of β-alanyl aminopeptidase enzyme activity; this contradicted the initial culture – MALDI-TOF-MS data which had not detected the presence of *P. aeruginosa*. However, further investigation of these ‘false positive’ samples (samples 6, 13, 16, 17, 27, 34 and 85) by further culturing, isolation and MALDI-TOF-MS confirmed the presence of a potentially β-alanyl aminopeptidase positive organism that had been originally unreported by the routine culture protocol for CF patient samples. The subsequent analyses identified the β-alanyl aminopeptidase activity as being attributable to *Serratia marcescens* (sample 27), *P. aeruginosa* (samples 6, 16, 17 and 85), and a *Pseudomonas* strain (sample 13 and 34); the latter could only be identified to genus level *via* MALDI-TOF-MS. All organisms isolated from the re-cultured samples were then tested as pure colonies using 3-amino-*N*-(3-fluorophenyl)propanamide and BHI broth as the growth medium, and all were re-confirmed to be positive for β-alanyl aminopeptidase activity.

**Table 3:** Comparison of exogenous volatile organic compound analysis by HS-SPME-GC-MS with culture – MALDI-TOF-MS of sputum samples from cystic fibrosis patients

Sample ID number	Patient gender	Patient age	Organisms identified from culture *via* MALDI-TOF-MS	3-Fluoroaniline concentration^a^ (μg mL^−1^)		Notes
				8 h incubation	24 h incubation	
1	M	51	*Candida* sp., ***P. fluorescens***, *S. aureus*	**3.0 (2.8, 3.2)**	**23.7 (22.9, 24.5)**	b
2	F	10	*Candida* sp., *E. dermatitidis*	0.0 (0.0, 0.0)	0.0 (0.0, 0.0)	
3	F	9	*E. dermatitidis*, *P. mirabilis*	0.0 (0.0, 0.0)	0.0 (0.0, 0.0)	
4	F	9	*Candida* sp., *E. dermatitidis*, *P. mirabilis*	0.0 (0.0, 0.0)	0.0 (0.0, 0.0)	
5	F	9	*A. fumigatus*, *E. dermatitidis*, *P. mirabilis*	0.0 (0.0, 0.0)	0.0 (0.0, 0.0)	
6	F	9	*E. coli*, *E. dermatitidis*, *P. mirabilis*, *S. maltophilia*, ***P. aeruginosa***	**0.5 (0.5, 0.5)**	**0.8 (0.8, 0.8)**	c
7	F	9	*E. dermatitidis*, *P. mirabilis*, *S. maltophilia*	0.0 (0.0, 0.0)	0.0 (0.0, 0.0)	
8	F	9	*A. fumigatus*, *Candida* sp., *E. dermatitidis*, *P. mirabilis*	0.0 (0.0, 0.0)	0.0 (0.0, 0.0)	
9	M	17	*A. fumigatus*, ***Serratia* sp.**	0.0 (0.0, 0.0)	0.0 (0.0, 0.0)	
10	F	11	*A. fumigatus*, *E. dermatitidis*	0.0 (0.0, 0.0)	0.0 (0.0, 0.0)	
11	F	11	*A. fumigatus*, *E. dermatitidis*, ***P. aeruginosa***	**0.3 (0.3, 0.3)**	**33.7 (33.5, 33.9)**	b
12	M	19	*A. fumigatus*, *E. dermatitidis*, *M. abscessus* complex	0.0 (0.0, 0.0)	0.0 (0.0, 0.0)	
13	M	19	*A. fumigatus*, *Candida* sp., *E. dermatitidis*, ***Pseudomonas* sp.**	**0.6 (0.7, 0.6)**	**2.5 (2.5, 2.6)**	d
14	M	7	*Candida* sp.	0.0 (0.0, 0.0)	0.0 (0.0, 0.0)	
15	M	7	*Candida* sp., ***P. aeruginosa***, ***Serratia* sp.**	**0.4 (0.4, 0.4)**	**1.7 (1.7, 1.7)**	b
16	F	14	*Candida* sp., *S. apiospermum*, ***P. aeruginosa***	0.0 (0.0, 0.0)	**0.4 (0.4, 0.4)**	c
17	F	14	*S. apiospermum*, ***P. aeruginosa***	0.0 (0.0, 0.0)	**4.0 (3.9, 4.2)**	c
18	F	14	*Candida* sp., ***P. aeruginosa***, *S. apiospermum*	**5.1 (4.8, 5.4)**	**8.0 (8.3, 7.8)**	b
19	M	24	*S. aureus*	0.0 (0.0, 0.0)	0.0 (0.0, 0.0)	
20	M	24	*A. fumigatus*, ***P. aeruginosa***, *S. aureus*	**0.6 (0.5, 0.6)**	**1.5 (1.5, 1.5)**	b
21	M	7	*Candida* sp., *E. coli*, *E. dermatitidis*	0.0 (0.0, 0.0)	0.0 (0.0, 0.0)	
22	M	27	*A. fumigatus*, *S. aureus*, *S. maltophilia*	0.0 (0.0, 0.0)	0.0 (0.0, 0.0)	
23	F	13	*Achromobacter* sp., *E. dermatitidis*	0.0 (0.0, 0.0)	0.0 (0.0, 0.0)	
24	F	13	*Achromobacter* sp., *E. dermatitidis*	0.0 (0.0, 0.0)	0.0 (0.0, 0.0)	
25	F	13	*Achromobacter* sp., *E. dermatitidis*	0.0 (0.0, 0.0)	0.0 (0.0, 0.0)	
26	F	13	*Achromobacter* sp., *Candida* sp., *E. dermatitidis*	0.0 (0.0, 0.0)	0.0 (0.0, 0.0)	
27	M	10	*Candida* sp., *E. miricola*, *E. dermatitidis*, ***S. marcescens***, *M. abscessus* complex	0.0 (0.0, 0.0)	**3.0 (3.0, 3.0)**	e
28	M	10	*Candida* sp., *E. dermatitidis*	0.0 (0.0, 0.0)	0.0 (0.0, 0.0)	
29	M	10	*Candida* sp.	0.0 (0.0, 0.0)	0.0 (0.0, 0.0)	
30	M	10	*Candida* sp.	0.0 (0.0, 0.0)	0.0 (0.0, 0.0)	
31	F	16	*Candida* sp., *E. dermatitidis*, ***Serratia* sp.**, *S. maltophilia*	**1.7 (1.4, 1.9)**	**4.8 (4.7, 5.0)**	b
32	F	16	*Candida* sp., *E. dermatitidis*, ***Serratia* sp.**, *M. llatzerense*	**2.2 (2.0, 2.1)**	**3.7 (3.7, 3.6)**	b
33	F	17	***P. aeruginosa***	**6.5 (7.0, 6.0)**	**28.5 (26.6, 30.3)**	b
34	F	17	***Pseudomonas* sp.**	**8.7 (8.8, 8.6)**	**14.8 (14.6, 15.1)**	d
35	F	17	*A. fumigatus*, ***P. aeruginosa***	**1.7 (1.6, 1.8)**	**5.4 (5.2, 5.6)**	b
36	M	32	***B. multivorans***, *Candida* sp., *S. aureus*, *S. maltophilia*	**0.4 (0.4, 0.4)**	**2.0 (2.0, 2.1)**	b
37	F	23	*Pandoraea* sp., *M. abscessus* complex	0.0 (0.0, 0.0)	0.0 (0.0, 0.0)	
38	M	24	***P. aeruginosa***	**2.2 (2.0, 2.4)**	**5.4 (5.2, 5.7)**	b
39	F	27	***P. aeruginosa***	**9.5 (8.9, 10.1)**	**17.9 (17.1, 18.7)**	b
40	F	27	***P. aeruginosa***	**4.0 (3.7, 4.3)**	**13.5 (13.5, 13.6)**	b
41	F	32	***P. aeruginosa***, *S. aureus*	**3.6 (3.6, 3.7)**	**7.1 (6.7, 7.4)**	b
42	F	34	*A. fumigatus*, *Candida* sp., ***P. aeruginosa***	**0.3 (0.3, 0.3)**	**5.3 (5.6, 5.1)**	b
43	M	38	*A. fumigatus*, ***B. multivorans***, *Candida* sp., *S. maltophilia*, *M. chelonae* complex	**0.3 (0.3, 0.3)**	**0.7 (0.7, 0.7)**	b
44	M	31	*Candida* sp., ***P. aeruginosa***	**2.7 (2.6, 2.9)**	**22.6 (21.8, 23.4)**	b
45	F	49	*Candida* sp., *Pandoraea* sp., ***P. aeruginosa***	**0.3 (0.3, 0.3)**	**0.5 (0.5, 0.5)**	b
46	F	65	*Achromobacter* sp.	0.0 (0.0, 0.0)	0.0 (0.0, 0.0)	
47	M	33	*Candida* sp., ***P. aeruginosa***, *S. maltophilia*	**2.5 (2.3, 2.6)**	**5.1 (5.0, 5.2)**	b
48	M	31	***B. multivorans***, *Candida* sp., ***P. aeruginosa***	**2.8 (2.7, 3.0)**	**4.8 (4.5, 5.1)**	b
49	F	8	N/A	0.0 (0.0, 0.0)	0.0 (0.0, 0.0)	
50	F	24	*Candida* sp., *S. agalactiae* (GpB)	0.0 (0.0, 0.0)	0.0 (0.0, 0.0)	
51	M	29	*E. coli*, ***P. aeruginosa***, *M. llatzerense*, *M. chelonae* complex	**2.9 (2.9, 2.9)**	**3.7 (3.7, 3.7)**	b
52	M	22	*E. dermatitidis*, ***P. aeruginosa***, *M. abscessus* complex	**0.3 (0.3, 0.4)**	**0.5 (0.5, 0.5)**	b
53	M	35	***B. multivorans***, *Candida* sp., ***P. aeruginosa***	**0.4 (0.3, 0.4)**	**1.0 (0.9, 1.1)**	b
54	M	8	*Neisseria elongata*	0.0 (0.0, 0.0)	0.0 (0.0, 0.0)	
55	F	19	*A. fumigatus*, *S. aureus*	0.0 (0.0, 0.0)	0.0 (0.0, 0.0)	
56	M	29	*Candida* sp., ***P. aeruginosa***	**7.8 (7.9, 7.8)**	**9.3 (9.2, 9.4)**	b
57	F	30	*Achromobacter* sp., ***P. aeruginosa***	**4.7 (4.4, 5.0)**	**8.2 (8.0, 8.4)**	b
58	M	62	N/A	0.0 (0.0, 0.0)	0.0 (0.0, 0.0)	
59	F	46	***P. aeruginosa***	**13.5 (12.8, 14.1)**	**27.9 (28.5, 27.2)**	b
60	M	42	*A. fumigatus*, *Candida* sp., ***P. aeruginosa***, *S. aureus*	**0.4 (0.3, 0.4)**	**0.7 (0.7, 0.7)**	b
61	F	40	*Candida* sp., ***P. aeruginosa***, *M. llatzerense*	**5.7 (5.5, 5.9)**	**21.0 (20.6, 21.4)**	b
62	F	44	***P. aeruginosa***	**4.0 (3.8, 4.3)**	**10.9 (10.9, 10.9)**	b
63	F	14	***P. aeruginosa***	**14.6 (14.9, 14.4)**	**22.3 (21.8, 22.7)**	b
64	M	49	*A. fumigatus*, *Candida* sp., *S. maltophilia*	0.0 (0.0, 0.0)	0.0 (0.0, 0.0)	
65	M	35	*A. fumigatus*, *Candida* sp., ***P. aeruginosa***	**8.2 (8.1, 8.3)**	**25.4 (25.8, 25.0)**	b
66	M	38	***P. aeruginosa***	**4.3 (4.2, 4.3)**	**17.0 (16.6, 17.4)**	b
67	M	12	N/A	0.0 (0.0, 0.0)	0.0 (0.0, 0.0)	
68	F	48	*Candida* sp., *S. aureus*	0.0 (0.0, 0.0)	0.0 (0.0, 0.0)	
69	M	36	*Candida* sp., ***P. aeruginosa***	**6.7 (6.6, 6.9)**	**21.8 (22.1, 21.5)**	b
70	F	24	*A. fumigatus*, *E. dermatitidis*, *H. influenzae*, *S. aureus*	0.0 (0.0, 0.0)	0.0 (0.0, 0.0)	
71	M	19	***P. aeruginosa***, *M. abscessus* complex	**6.3 (6.3, 6.2)**	**9.1 (8.8, 9.5)**	b
72	M	23	*Candida* sp., ***P. aeruginosa***, *S. maltophilia*, *M. abscessus* complex	**0.6 (0.6, 0.6)**	**1.1 (1.1, 1.1)**	b
73	M	31	*E. dermatitidis*, ***P. aeruginosa***	**12.7 (12.9, 12.5)**	**22.5 (22.7, 22.3)**	b
74	M	34	*Candida* sp., *S. aureus*	0.0 (0.0, 0.0)	0.0 (0.0, 0.0)	
75	F	14	*A. fumigatus*, *Candida* sp., *E. dermatitidis*, ***P. aeruginosa***	**0.4 (0.4, 0.4)**	**1.0 (1.1, 1.0)**	b
76	M	37	*Achromobacter* sp.	0.0 (0.0, 0.0)	0.0 (0.0, 0.0)	
77	M	29	*Candida* sp., *E. dermatitidis*, *Rothia* sp., *S. maltophilia*	0.0 (0.0, 0.0)	0.0 (0.0, 0.0)	
78	F	70	*Candida* sp., ***P. aeruginosa***, *S. aureus*	**0.3 (0.3, 0.3)**	**0.5 (0.6, 0.5)**	b
79	M	25	*Candida* sp., *E. dermatitidis*, ***P. aeruginosa***, *Rothia* sp.	**0.3 (0.3, 0.3)**	**5.5 (5.4, 5.7)**	b
80	M	24	***P. aeruginosa***, *M. abscessus* complex	**0.9 (0.9, 1.0)**	**25.0 (24.6, 25.5)**	b
81	F	21	*Achromobacter* sp., *Candida* sp., ***P. aeruginosa***	**0.5 (0.5, 0.5)**	**1.2 (1.1, 1.2)**	b
82	F	27	*Candida* sp., *H. influenzae*, ***P. aeruginosa***, ***Serratia* sp.**, *S. aureus*	**2.7 (2.6, 2.8)**	**4.6 (4.5, 4.7)**	b
83	F	35	*Candida* sp., *K. oxytoca*, ***P. aeruginosa***, *S. aureus*	**0.3 (0.3, 0.3)**	**1.2 (1.1, 1.2)**	b
84	F	39	***B. multivorans***, *Candida* sp., *S. maltophilia*	**0.3 (0.3, 0.4)**	**0.7 (0.7, 0.8)**	b
85	M	9	***P. aeruginosa***	**24.5 (24.7, 24.2)**	**32.9 (34.1, 31.7)**	c
86	F	26	*A. fumigatus*, *S. aureus*	0.0 (0.0, 0.0)	0.0 (0.0, 0.0)	
87	F	66	*Candida* sp., *S. aureus*	0.0 (0.0, 0.0)	0.0 (0.0, 0.0)	
88	M	32	*Candida* sp., *S. aureus*	0.0 (0.0, 0.0)	0.0 (0.0, 0.0)	
89	M	47	*A. fumigatus*, *S. maltophilia*, *M. abscessus* complex	0.0 (0.0, 0.0)	0.0 (0.0, 0.0)	
90	F	18	*A. fumigatus*, *Candida* sp., ***P. aeruginosa***	**4.7 (4.6, 4.8)**	**24.4 (33.9, 34.8)**	b
91	M	21	***B. multivorans***, *Candida* sp., ***P. aeruginosa***, ***Serratia* sp.**	**2.1 (2.1, 2.1)**	**8.5 (7.0, 9.9)**	b
92	F	25	*Candida* sp., ***P. aeruginosa***	**2.7 (2.6, 2.8)**	**4.5 (4.3, 4.7)**	b
93	F	26	*Candida* sp., ***P. aeruginosa***	**7.8 (7.6, 8.1)**	**8.3 (7.9, 8.7)**	b
94	F	49	*Candida* sp., *S. aureus*	0.0 (0.0, 0.0)	0.0 (0.0, 0.0)	
95	M	35	*Candida* sp., ***P. aeruginosa***	**0.5 (0.4, 0.5)**	**1.3 (1.4, 1.2)**	b
96	M	34	*S. aureus*	0.0 (0.0, 0.0)	0.0 (0.0, 0.0)	
97	F	22	*Candida* sp., ***P. aeruginosa***	**0.3 (0.3, 0.3)**	**0.7 (0.7, 0.8)**	b
98	M	47	*Candida* sp., ***P. aeruginosa***	**1.9 (2.0, 1.7)**	**3.8 (3.7, 4.0)**	b
99	M	NR	*Achromobacter* sp.	0.0 (0.0, 0.0)	0.0 (0.0, 0.0)	
100	M	NR	*Candida* sp.	0.0 (0.0, 0.0)	0.0 (0.0, 0.0)	
101	F	18	***P. aeruginosa***	**7.1 (6.9, 7.4)**	**26.4 (26.9, 26.0)**	b ^,^ f
102	M	38	*Candida* sp., *S. aureus*	0.0 (0.0, 0.0)	0.0 (0.0, 0.0)	f
103	F	37	*Achromobacter* sp., *A. fumigatus*, ***P. aeruginosa***	**17.0 (17.5, 16.5)**	**23.1 (26.2, 20.1)**	b ^,^ f
104	M	28	*Achromobacter* sp., ***P. aeruginosa***	**6.1 (6.5, 5.8)**	**8.4 (8.7, 8.1)**	b ^,^ f
105	F	20	*Candida* sp., *S. aureus*	0.0 (0.0, 0.0)	0.0 (0.0, 0.0)	f

a Mean (individual value).

b β-Alanine activity detected + causative organism identified in routine culture testing.

c *P. aeruginosa* initially missed by routine culture; positive β-alanine activity determined following subsequent pure isolate testing.

d *Pseudomonas* sp. initially missed by routine culture; positive β-alanine activity determined following subsequent pure isolate testing.

e *S. marcescens* initially missed by routine culture; positive β-alanine activity determined following subsequent pure isolate testing.

f Post lung transplant sample. NR = not recorded. NA = not applicable; no organisms detected.

Respiratory infections caused by *P. aeruginosa* are very common in patients with CF and once chronic infection is established, it is almost impossible to eradicate and is associated with increased mortality and morbidity.^17^ If infection can be detected early, there is deemed to be a ‘window of opportunity’ for treatment with antibiotics that can result in eradication that is sustained for up to two years.^17^ Isolation of *P. aeruginosa* using standard culture methods is not always straightforward as the lung airways of patients with CF may contain several dozen distinct bacterial species and well as fungi^38^ that can compete with *P. aeruginosa* to form detectable colonies on culture media. Isolation of colonies by culture can be particularly challenging if the abundance of *P. aeruginosa* is very low, relative to other co-inhabitants of the lung. This is likely to be the case when *P. aeruginosa* first colonizes the airways prior to chronic colonization and infection. To circumvent this problem, Billard-Pomares *et al.* developed a quantitative polymerase chain reaction (PCR) method to detect *P. aeruginosa* in sputum samples and demonstrated a detection limit of around 15 CFU mL^−1^ of sputum.^39^ Using this highly-sensitive method, they were able to demonstrate detection of *P. aeruginosa* in 10/33 sputum samples that were negative using standard culture methods. They concluded that this method may be a very useful tool in association with bacterial culture for the diagnosis of early *P. aeruginosa* colonization and the monitoring of *P. aeruginosa* eradication after antimicrobial therapy in young CF patients.^39^ One limitation of PCR methods, as highlighted by the authors, was the possibility that dead or non-viable *P. aeruginosa* would be detected and this could be particularly problematic if PCR is used to monitor the outcome of eradication therapy – which is another scenario where the bacterial load of *P. aeruginosa* may be low and more challenging to detect using standard culture methods.

## Conclusions

To the best of our knowledge, this is the first demonstration of detection of *P. aeruginosa* by use of a custom-designed substrate to liberate a detectable VOC. The test includes a selective enrichment broth that can potentially accommodate a high volume of sputum resulting in high sensitivity. In comparison, with standard culture methods, this new culture method demonstrated 100% sensitivity and resulted in the detection of six additional isolates of *Pseudomonas* species (four of which were confirmed as *P. aeruginosa*) that were initially missed by culture. The very high negative predictive value (100% in this study) means that such an assay could be appropriate as a screening technique for patients who are not yet colonized by this pathogen. Future work will focus on a simplified means for detection of the VOC liberated by β-alanyl aminopeptidase activity (*e.g.* using a colorimetric indicator) to obviate the requirement for sophisticated instrumentation.

## Conflicts of interest

There are no conflicts of interest.

## Supplementary Material
